# Temporal-Enhanced and Visual-Text Adaptive Fusion for Weakly Supervised Video Anomaly Detection in Public Safety

**DOI:** 10.3390/jimaging12060249

**Published:** 2026-06-06

**Authors:** Jin Si, Qifen Dong, Xue Yang

**Affiliations:** 1 Big Data and Network Security Research Institute, Zhejiang Police College, Hangzhou 310053, China; 2 School of Information and Network Security, Zhejiang Police College, Hangzhou 310053, China; dongqifen@zjjcxy.cn (Q.D.); yangxue@zjjcxy.cn (X.Y.)

**Keywords:** anomaly detection, weakly supervised learning, multiple instance learning, I3D, visual-text model

## Abstract

In the realm of public safety, the automated identification of potential threats from voluminous surveillance streams is pivotal for developing intelligent security systems. Manual monitoring of such massive video feeds is highly inefficient, prone to human fatigue, and often leads to missed detections or false alarms. Leveraging deep learning for automatic anomaly detection is therefore essential to improve response efficiency and mitigate security risks. Weakly supervised video anomaly detection (WS-VAD) has emerged as a critical yet challenging task in this domain. In this study, we propose the Temporal-Enhanced and Visual-Text Adaptive Fusion (TE-VTAF) model for robust WS-VAD. Specifically, a Dynamic Local–Global Temporal Adaptive Module (DLG-TAM) is designed to capture multi-scale temporal dependencies and extract high-level video semantics. Concurrently, a Visual-Text Adaptive Fusion Module (VTAFM) is introduced to aggregate complementary cross-modal features, utilizing a competitive activation mechanism to suppress redundant information and enhance the discriminative power between normal and anomalous events. To further refine the learning process within the Multiple Instance Learning (MIL) framework, we incorporate a Top-K outer bag loss and a K-maxmin inner bag loss. These constraints effectively maximize the inter-class separability while suppressing label noise from normal instances within positive bags, thereby bolstering the detector’s robustness. Extensive experiments demonstrate that the proposed TE-VTAF consistently outperforms state-of-the-art methods on two large-scale benchmarks, achieving an AUC of 88.93% on UCF-Crime and an AP of 85.62% on XD-Violence.

## 1. Introduction

Video anomaly detection in public safety aims to automatically identify potential threat events—such as violence, sudden incidents, illegal intrusions, and abnormal gatherings—from large-scale unedited surveillance videos. This capability represents one of the core technologies for constructing intelligent urban security monitoring systems. The task is challenged by the extreme sparsity, diversity, and unpredictability of anomalous events. In critical areas such as public spaces, transportation hubs, campuses, and airports, manual monitoring of massive video streams is inefficient and costly and often results in missed detections or false alarms due to operator fatigue. Therefore, leveraging deep learning techniques for automatic anomaly detection is crucial for improving response efficiency and mitigating potential security risks.

In weakly supervised video anomaly detection (WS-VAD), anomaly detectors require only coarse-grained video-level annotations to produce frame-level or snippet-level anomaly scores via mechanisms such as multiple instance learning (MIL). This paradigm significantly reduces annotation costs while enabling spatiotemporal localization of anomalous events. Early approaches typically employ pre-trained C3D [1] or I3D [2] models to extract spatiotemporal features, and learn to discriminate between normal and anomalous snippets within a multiple instance learning (MIL) framework [3,4,5,6], thereby achieving anomaly localization under weak supervision. However, these methods primarily rely on visual spatiotemporal features and lack the capability to capture high-level semantic information. More importantly, they overlook the cross-modal relationships between visual content and semantic descriptions, which limits their ability to recognize anomalous events with semantic ambiguity or strong contextual dependency in complex scenarios.

Driven by recent advancements in vision-language pre-training [7], Contrastive Language-Image Pre-training (CLIP) [8] has catalyzed a paradigmatic shift in weakly supervised video anomaly detection (WS-VAD), leveraging its robust cross-modal semantic alignment capabilities. By harnessing extensive semantic knowledge acquired through large-scale image-text contrastive learning, CLIP facilitates the mapping of latent correspondences between visual representations and textual semantics, thereby opening novel methodological avenues for the semantic interpretation of anomalous events. Consequently, several recent studies have integrated CLIP into the WS-VAD framework [9,10,11]. Despite these strides, existing CLIP-based methodologies exhibit several critical limitations. Firstly, most approaches rely on static text prompts or rudimentary temporal aggregation, which hampers their ability to simultaneously capture short-term local variations, long-range global dependencies, and multi-scale temporal evolutions. In particular, they struggle to model anomalies with large temporal span variations or short-term interleaving patterns (e.g., theft following prolonged loitering), often introducing background noise or causing attention dispersion, thereby leading to weak temporal representations of anomalies [12,13]. Secondly, current visual-text fusion strategies frequently employ simplistic concatenation or summation, which tends to generate information redundancy and fails to adaptively discern subtle anomalies within complex environments [14]. Furthermore, conventional ranking or Multiple Instance Learning (MIL) losses focus exclusively on the most salient instances. This narrow focus overlooks the potential of multiple anomalous snippets within a single video and provides inadequate suppression of label noise from normal snippets in positive bags. By disregarding the underlying temporal structure of events, these methods often suffer from suboptimal training efficiency, ambiguous anomaly boundaries, and elevated false positive rates [15].

To fully harness the cross-modal semantic knowledge of CLIP and unlock its greater potential in weakly supervised video anomaly detection, several critical challenges must be addressed. First, a dynamic temporal adaptation mechanism is required that can simultaneously capture short-term local abrupt changes and long-range global semantic dependencies among video snippets, thereby enhancing the temporal representational power of anomalous events. Second, a competitive adaptive fusion strategy must be constructed between visual features and textual semantic priors to fully aggregate complementary cross-modal information while effectively suppressing redundancy, thus strengthening the semantic expressiveness of subtle anomalies in complex scenes. Finally, under weakly supervised settings, an optimization loss function should be introduced that fully exploits the temporal structure of multiple anomalous snippets within anomaly videos and substantially mitigates noise interference from normal instances inside positive bags, thereby improving training efficiency and enabling more precise normal/abnormal boundary separation.

To circumvent the aforementioned limitations, we propose a Temporal-Enhanced and Visual-Text Adaptive Fusion (TE-VTAF) framework for WS-VAD in public safety applications. The proposed framework consists of three key components. To tackle the first challenge, we design a Dynamic Local–Global Temporal Adaptive Module (DLG-TAM), which captures both short-term local variations and long-range semantic dependencies across video snippets. Specifically, this module integrates a local selective temporal unit with a global temporal distance-aware unit, enabling the model to effectively model multi-scale temporal dynamics and better characterize complex anomaly evolution patterns. To address the second challenge, we introduce a Visual-Text Adaptive Fusion Module (VTAFM) to establish more effective cross-modal interactions between visual features and textual semantic priors. By incorporating cross-modal attention and adaptive fusion mechanisms, VTAFM dynamically balances the contributions of visual and textual information, thereby enhancing semantic representations while suppressing redundant and noisy cues in complex environments. Finally, to improve the training process, we propose a novel MIL-based objective function consisting of a Top-K outer bag loss and a K-maxmin inner bag loss. The Top-K outer bag loss focuses on the top-k highest-scoring instances, enabling the model to fully exploit multiple anomalous snippets within anomalous videos. Meanwhile, the K-maxmin inner bag loss leverages the inherent temporal structure of anomalous and normal events by reshaping the distribution of anomaly scores within each video. This design effectively suppresses noise from normal snippets in anomalous videos while maximizing the separation between normal and anomalous snippets.

The contributions in this work are summarized as follows:

(1) We propose a Temporal-Enhanced and Visual-Text Adaptive Fusion (TE-VTAF) framework for WS-VAD in public safety. Through a Dynamic Local–Global Temporal Adaptive Module (DLG-TAM), the framework effectively models both short-term dynamic variations and long-range global semantic dependencies among video snippets, thereby enhancing the temporal representational power of anomalous events.

(2) We propose a Visual-Text Adaptive Fusion Module (VTAFM) that employs cross-modal cross-attention and attention-based feature fusion mechanisms to achieve dynamic weight allocation (i.e., competitive fusion) between visual features and textual semantic priors. This enables the aggregation of complementary cross-modal information while effectively suppressing redundancy, thereby strengthening the semantic expressiveness of anomalous events in complex scenes.

(3) Building upon existing ranking losses, we introduce a Top-K outer bag loss and a K-maxmin inner bag loss. These loss functions exploit the latent temporal structure of both anomalous and normal events, maximally leveraging multiple anomalous snippets potentially present in anomaly videos for effective training while mitigating noise interference from normal snippets within anomalous videos.

(4) The proposed method outperforms state-of-the-art (SOTA) approaches on two benchmark datasets and exhibits superior robustness.

## 2. Related Works

### 2.1. Weakly Supervised Video Anomaly Detection

Video anomaly detection methods are typically categorized into three groups based on the granularity and type of supervision: unsupervised/one-class classification, weakly supervised, and fully supervised approaches. Compared with unsupervised methods that model only normal data and fully supervised methods that require expensive frame-level annotations, weakly supervised video anomaly detection relies solely on video-level labels to explicitly model anomalous patterns, achieving a favorable balance between detection performance and annotation cost.

Sultani et al. [3] were the first to introduce multiple instance learning (MIL) into this task, treating anomalous and normal videos as positive and negative bags, respectively, and learning snippet-level anomaly scores via a deep ranking framework. Building upon this formulation, subsequent studies have improved the MIL framework from the perspectives of intra-bag constraints and feature representation. For instance, Zhang et al. [4] introduced an intra-bag loss to strengthen instance-level constraints, while Zhu et al. [5] proposed an attention-based MIL ranking model to learn more discriminative temporal features. However, the presence of label noise within positive bags limits the effectiveness of these methods in leveraging anomaly annotations. To mitigate this issue, Zhong et al. [16] reformulated the problem as supervised learning with noisy labels and employed graph convolutional networks (GCNs) [17] for high-confidence instance selection. Zaheer et al. [18] reduced noise interference by generating pseudo-labels via clustering, whereas Feng et al. [19] proposed a multi-instance self-training framework to progressively refine pseudo-label quality.

Considering that anomalous events often exhibit continuous temporal structures, recent studies have increasingly focused on temporal modeling. Tian et al. [20] enhanced feature separability by selecting Top-k key snippets, while Li et al. [21] introduced a multi-sequence learning (MSL) framework combined with Transformer architectures [22] to capture long-range temporal dependencies. Furthermore, Chen et al. [23] leveraged video descriptions to generate textual features for video snippets and fused them with visual features to improve detection performance. However, such approaches are typically limited to static semantic alignment or shallow feature fusion, which restricts their ability to capture deep interactions between visual dynamics and linguistic semantics.

To overcome these limitations, recent efforts have begun to explore large vision-language models (LVLMs), aiming to develop more semantically robust anomaly detection paradigms by leveraging their superior cross-modal reasoning and open-world perception capabilities.

### 2.2. Vision-Language Learning in VAD

Driven by the ascendance of vision-language pre-training (VLP) models, researchers have increasingly sought to harness their formidable cross-modal semantic alignment capabilities for video anomaly detection (VAD). Departing from early approaches that relied on superficial textual features derived from video captions [23], contemporary literature leverages the contrastive pre-training knowledge of CLIP—trained on massive image-text pairs—to establish fine-grained correspondences between spatio-temporal visual dynamics and linguistic semantics. Wu et al. [24] pioneered the integration of CLIP into the weakly supervised VAD (WS-VAD) task, directly adapting the frozen CLIP backbone in a training-free manner. Their approach employs a dual-branch architecture that bifurcates into coarse-grained visual classification and language-aligned similarity computation to derive frame-level anomaly scores.

Building upon this foundation, subsequent studies have focused on refining temporal modeling, enhancing semantic alignment, and optimizing computational efficiency. Specifically, Wang et al. [25] introduced the RelVid framework, which utilizes an Adapter-based mechanism to capture temporal dependencies and incorporates auxiliary tasks, such as textual anomaly detection and feature reconstruction, to enlarge inter-class separability. Zanella et al. [11] proposed AnomalyCLIP, which reconfigures the CLIP latent space around normal prototypes and employs directional learning for joint detection and classification, facilitated by a Selector model and an Axial Transformer for semantic-guided MIL. Addressing efficiency, Zhu et al. [26] developed ProDisc-VAD, which achieves state-of-the-art performance with minimal parameter overhead via a prototype interaction layer and extreme instance contrastive learning. Furthermore, to improve cross-domain generalization, Biswas et al. [27] introduced MMVAD, utilizing adaptive long-short-term snippetation and attention-based fusion for robust detection across UAV and CCTV environments. Notwithstanding these advancements in cross-modal alignment, the potential of CLIP-based methods for robust temporal dynamic modeling and the suppression of label noise within positive bags remains under-explored. Addressing these deficiencies constitutes the primary focus of this study.

## 3. The Proposed Method

**Figure 1:**
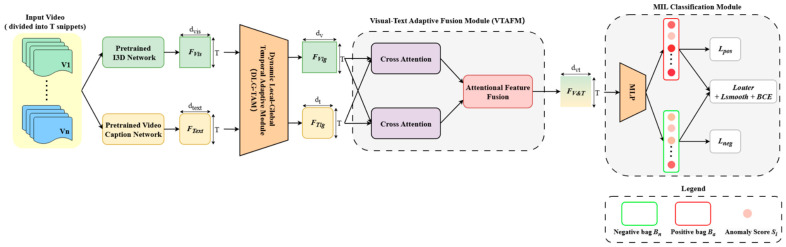
The overall architecture of the proposed Temporal-Enhanced and Visual–Text Adaptive Fusion (TE-VTAF) framework.

In this section, we present the overall architecture of the Temporal-Enhanced and Visual-Text Adaptive Fusion (TE-VTAF) network, as shown in Figure 1. First, we introduce the DLG-TAM (Dynamic Local–Global Temporal Adaptation Module) to capture both short- and long-term temporal dependencies among snippet features (Section 3.1). Next, we propose the visual-text adaptive fusion module (VTAFM) to effectively integrate the perceptual strength of visual information in capturing dynamic scene details with the expressive power of textual information in representing abstract semantics, thereby optimizing the visual and textual feature representations of video content (Section 3.2). Finally, we incorporate Top-K outer bag loss and K-maxmin in-bag loss to optimize model training (Section 3.3).

### 3.1. Dynamic Local–Global Temporal Adaptive Module

**Figure 2:**
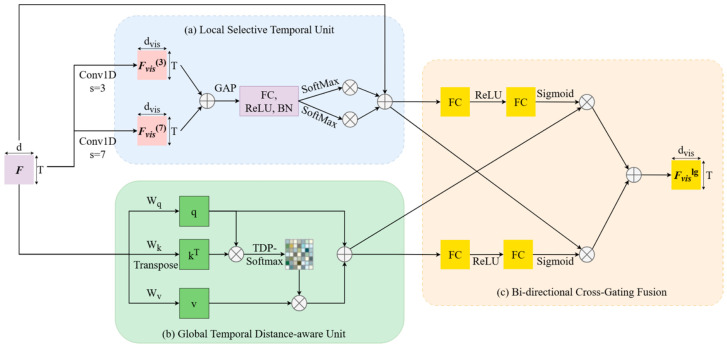
Architecture of the Dynamic Local–Global Temporal Adaptive Module, including (**a**) the Local Selective Temporal Unit (LSTU), (**b**) the Global Temporal Distance-aware Unit (GTDU), and (**c**) the Bi-directional Cross-Gating Fusion (BCGF) module.

The objective of weakly supervised video anomaly detection (WS-VAD) is to determine whether a video contains anomalous snippets and to accurately localize them. However, anomalous events often exhibit significant temporal scale variations and are easily affected by redundant background information. Consequently, modeling temporal dependencies across video snippets is critical for reliable anomaly detection. To simultaneously capture fine-grained local action variations and long-range semantic dependencies, we propose a Dynamic Local–Global Temporal Adaptive Module (DLG-TAM). As illustrated in Figure 1, the proposed module comprises three key sub-modules: a Local Selective Temporal Unit (LSTU), a Global Temporal Distance-aware Unit (GTDU), and a Bi-directional Cross-Gating Fusion (BCGF) branch. The overall network architecture is shown in Figure 2.

#### 3.1.1. Local Selective Temporal Unit

Anomalous events (e.g., fighting and robbery) typically exhibit temporal continuity, where visual features across adjacent snippets either undergo significant variations or follow specific temporal evolution patterns. Therefore, enhancing local temporal representations across adjacent snippets is critical for accurate anomaly detection. Adjacent snippets often exhibit strong semantic coherence, and aggregating local context enables the model to capture fine-grained dynamic variations. Moreover, local temporal aggregation helps suppress spurious noise present in isolated snippets, thereby improving the stability and reliability of snippet-level anomaly score prediction. However, conventional temporal convolutional layers employ fixed kernel sizes, limiting their ability to adapt to the diverse temporal patterns of anomalous events. To address this limitation, we propose a Local Selective Temporal Unit (LSTU), which extracts multi-scale features via parallel convolutional branches with different kernel sizes. Furthermore, a channel-attention-based dynamic guidance mechanism is introduced to adaptively adjust the receptive field, thereby effectively enhancing local temporal dependencies across adjacent snippets.

As both visual and textual features are processed by this module, we take the visual feature $F_{Vis}\in R^{T\times d_{vis}}$ of video V as an illustrative example to describe the overall architecture. Specifically, a 1D convolutional layer with a kernel size of s∈{3,7} is applied to the video features, which is formally defined as

(1) $$F_{vis}^{\left(s\right)}={Conv1D}^{\left(s\right)}(F_{Vis})$$

where ${Conv1D}^{\left(s\right)}(\cdot )$ denotes a 1D convolution operation with kernel size of s, and $F_{vis}^{\left(s\right)}\in R^{T\times d_{vis}}$ represents the resulting feature obtained by applying the convolution to the input video feature $F_{vis}^{\left(s\right)}$ along the temporal dimension.

Subsequently, the features $F_{vis}^{\left(3\right)}$ and $F_{vis}^{\left(7\right)}$ are fused via element-wise addition, followed by global average pooling (GAP) and a fully connected (FC) layer to produce a channel-wise descriptor. A Softmax function is then applied to compute the selection weights $w_{3}$ and $w_{7}$ for the two branches:

(2) $$w_{3},w_{7}=SoftMax(FC(GAP(F_{vis}^{\left(3\right)}+F_{vis}^{\left(7\right)})))\;w_{3}+w_{7}=1$$

where SoftMax(·) denotes the Softmax function, FC(·) denotes a fully connected (linear) layer, and GAP(·) denotes global average pooling. The final locally enhanced feature $F_{vis}^{local}$ is obtained via attention-based dynamic weighting:

(3) $$F_{\mathrm{vis}}^{\mathrm{local}}=⊗F_{vis}^{\left(3\right)}+w_{7}⊗F_{vis}^{\left(7\right)}+F_{\mathrm{Vis}}$$

where ⊗ denotes channel-wise element-wise multiplication. In a data-driven manner, the model adaptively selects the most appropriate resolution during inference based on the complexity of the action. When encountering abrupt anomalies, the model tends to assign greater importance to the small-kernel branch to preserve fine-grained temporal details; in contrast, for gradually evolving anomalies, it leverages the large-kernel branch to capture more complete temporal structures of the action.

#### 3.1.2. Global Temporal Distance-Aware Unit

The challenge of weakly supervised video anomaly detection (WS-VAD) extends beyond identifying isolated anomalous events, requiring a deeper understanding of complex event logic and temporal relationships. Many anomalies (e.g., theft following prolonged loitering) span long temporal durations. Moreover, certain snippets that appear normal in isolation (e.g., rapid running) can only be correctly interpreted within a long-range global context, such as whether they are associated with pursuit. Therefore, modeling only local temporal dependencies is insufficient. Capturing global temporal dependencies across snippets is essential for distinguishing normal and anomalous patterns and enhancing discriminative capability. Most existing methods employ standard self-attention mechanisms to compute feature similarity in the latent space across all snippet pairs, enabling global information interaction beyond local convolution. However, they overlook the intrinsic temporal structure of videos, which can lead to overestimated attention weights, causing attention diffusion and the introduction of irrelevant background noise. To address this issue, we propose a temporal distance-aware multi-head self-attention module, in which a temporal distance-based penalty (TDP) matrix $M_{dist}$ is incorporated into the attention weights. This design introduces a physically grounded prior: snippets that are closer in time are more likely to belong to the same continuous event with underlying causal relationships. Consequently, the proposed mechanism enables joint modeling of content similarity and temporal continuity.

Taking the original I3D feature $F_{Vis}\in R^{T\times d_{vis}}$ as input, we first generate the query matrix Q, key matrix K, and value matrix V via linear projections. The temporal distance-based penalty matrix $M_{dist}$ is then added to the attention score matrix, followed by a softmax operation to compute the attention weights. This process yields the final output $F_{vis}^{global}\in R^{T\times d_{vis}}$, which is formulated as follows:

(4) $$M_{dist}=\left|i-j\right|$$

(5) $$F_{vis}^{global}=SoftMax\{(\frac{Q\cdot K^{T}}{\sqrt{d_{k}}}-\gamma \cdot M_{dist})\cdot V\}+F_{Vis}$$

where i and j denote the temporal indices of snippets, and $d_{k}$ represents the dimensionality of Q and K. The hyperparameter γ controls the strength of the temporal penalty, suppressing redundant correlations between snippets that are feature-similar yet temporally distant. This not only effectively mitigates attention diffusion but also encourages the network to form more compact and semantically coherent action clusters in the feature space, yielding more discriminative and less noisy globally enhanced features.

#### 3.1.3. Bi-Directional Cross-Gating Fusion

To achieve deep integration of local and global temporal correlations, we further design a bi-directional cross-gating fusion branch (Bi-directional Cross-Gating Fusion, BCGF). Traditional multi-scale feature fusion typically relies on simple concatenation or element-wise addition. However, local temporal associations primarily capture sudden local motions and are highly sensitive to high-frequency noise such as abrupt illumination changes and local occlusions; in contrast, global temporal associations focus on macroscopic semantic representations and often contain substantial redundant background information. Simple linear summation fails to eliminate the inherent noise within each branch, resulting in information redundancy and semantic ambiguity in the feature space. To address these limitations, we propose a bi-directional cross-gating mechanism that leverages the macroscopic distribution of global information to filter redundancy in local features while utilizing the microscopic variations in local features to calibrate global features. The specific formulation is as follows:

(6) $$W_{l}=\sigma \left(FC\left(GAP\left(F_{vis}^{global}\right)\right)\right),\;W_{g}=\sigma (FC(GAP(F_{vis}^{local})))$$

(7) $$F_{vis}^{lg}=W_{l}\cdot F_{vis}^{local}⊕W_{g}\cdot F_{vis}^{global}$$

where σ(·) denotes the sigmoid function. This bidirectional adaptive fusion strategy ensures that the feature $F_{vis}^{lg}$ produced by DLG-TAM preserves fine-grained local temporal dependencies while simultaneously capturing long-range global temporal dependencies.

### 3.2. Visual-Text Adaptive Fusion Module, VTAFM

Visual features extract high-level semantic representations from videos by modeling appearance and spatiotemporal information. Incorporating temporal dependencies into these features significantly improves the detection performance. However, under complex scenarios—particularly when anomalous events involve severe occlusions, abrupt illumination changes, or when the anomalous actions themselves are not visually salient—reliance on visual cues alone may lead to overlooking critical anomaly-related information. Notably, these visually subtle and easily obscured cues are often essential for capturing deeper semantic representations in videos.

**Figure 3:**
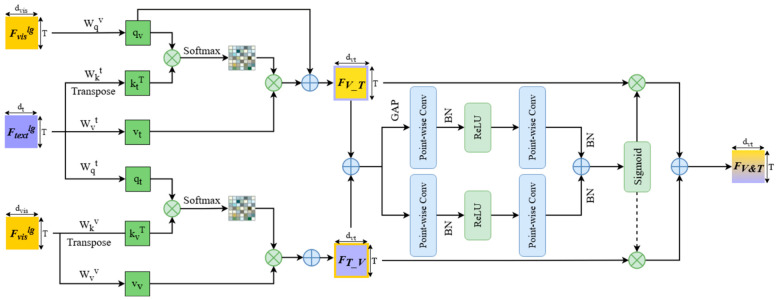
Architecture of the Visual–Text Information Competitive Fusion Module.

To overcome the limitations of single-modality representations in capturing video-level semantics, we propose a Visual-Text Adaptive Fusion Module (VTAFM), the detailed architecture of the proposed module is shown in Figure 3. This module incorporates a cross-attention mechanism and an Attentional Feature Fusion (AFF) module. The cross-attention mechanism bridges the modality gap, enabling the model to capture coherent visual dynamics while leveraging textual priors to ground abstract semantic concepts. Following cross-modal interaction, the AFF module addresses the limitations of static weight assignment by dynamically evaluating the relative importance of visual and textual modalities through global channel-wise context. This design enables nonlinear and adaptive fusion of heterogeneous features. As a result, the proposed module enhances the extraction of high-level semantic representations, reduces false positives and false negatives caused by complex scenes, and improves the robustness of detecting subtle anomalous events while maintaining high detection accuracy.

To introduce cross-modal semantic guidance, textual features are extracted using a frozen CLIP text encoder following the commonly adopted paradigm in existing CLIP-based WS-VAD methods. Specifically, manually predefined descriptive text templates are employed to construct textual inputs, without utilizing learnable prompts or dynamic prompt generation strategies based on external knowledge bases.

The textual prompts mainly describe coarse-grained normal or anomalous surveillance events. Representative prompts include: “A video of normal events”, “A video of anomalous events”, “A surveillance video containing abnormal behavior”, and “A surveillance video with violent or dangerous activities”.

These prompts remain fixed during both training and inference stages. The generated textual semantic embeddings are subsequently fused with visual snippet features through the proposed VTAFM to enhance anomaly-related semantic representation capability via cross-modal interaction. In addition, the CLIP text encoder remains frozen throughout the entire training process, while only the proposed temporal modeling and fusion modules are optimized.

To integrate textual information into visual features, the temporally enhanced visual features $F_{vis}^{lg}\in R^{T\times d_{vis}}$ and temporally enhanced textual features $F_{text}^{lg}\in R^{T\times d_{t}}$ are input to a cross-attention module. Specifically, $F_{vis}^{lg}$ and $F_{text}^{lg}$ are projected via linear projections to obtain the query matrix $Q_{v}$, key matrix $K_{t}$, and value matrix $V_{t}$. The cross-attention scores between text and visual features are then computed, followed by a residual connection, yielding the text-enhanced visual features $F_{V\_T}\in R^{T\times d_{vt}}$, which is formulated as follows:

(8) $$F_{V\_T}=CA\left(Q_{v},K_{t},\;V_{t}\right)=softmax\left(\frac{Q_{v}\cdot {K_{t}}^{T}}{\sqrt{d_{vt}}}\right)\cdot V_{t}+Q_{v}$$

where $d_{vt}$ denotes the dimensionality of $Q_{v}$ and $K_{t}$.

Subsequently, $F_{vis}^{lg}$ and $F_{text}^{lg}$ are fed into the cross-attention module, and through a similar projection and attention computation process, visually enhanced textual features $F_{T\_V}\in R^{T\times d_{vt}}$ are obtained, which are formulated as follows:

(9) $$F_{T\_V}=CA\left(Q_{t},K_{v},\;V_{v}\right)=softmax\left(\frac{Q_{t}\cdot {K_{v}}^{T}}{\sqrt{d_{tv}}}\right)\cdot V_{v}+Q_{t}$$

where $d_{tv}$ denotes the dimensionality of $Q_{t}$ and $K_{v}$.

Upon establishing the inter-modal interactive guidance, the two resulting features are fed into the Attentional Feature Fusion (AFF) module. This module concurrently aggregates local and global channel contexts to mitigate the inherent discrepancies in semantics and scales between cross-modal features.

Firstly, the fusion process commences with an initial integration of the two interactively guided features via element-wise summation:

(10) $$X\;=\;F_{V\_T}{⊕F}_{T\_V}$$

Local Channel Context branch: applies pointwise (1 × 1) convolution to perform channel interactions across spatial locations, thereby preserving fine-grained visual details that are sensitive to small-scale anomalies:

(11) L(X)=BN(PWConv2(δ(BN(PWConv1(X)))))

Global Channel Context branch: applies GAP to aggregate spatial information and capture the global semantic distribution of the video, followed by channel-wise dimensionality reduction and expansion to model inter-channel dependencies:

(12) g(X)=BN(PWConv2(δ(BN(PWConv1(GAP(X))))))

where PWConv1 and PWConv2 denote the reduction and expansion pointwise convolutions with a reduction ratio r, respectively. BN denotes batch normalization, and δ represents the ReLU activation function.

The local and global channel contexts are combined via broadcasting addition, followed by a sigmoid activation to generate a fusion weight matrix *M*(*X*), which is then used to perform complementary soft weighting on the interaction features through adaptive reweighting.

(13) MX=σ(L(X)+g(X))

(14) $$F_{V\&T}=M(X)\cdot F_{V\_T}+(1-M(X))\cdot F_{T\_V}$$

This dynamic balancing mechanism based on MS-CAM enables the VTAFM to achieve fine-grained feature allocation between visual-driven features and text-prior-enhanced representations. When encountering potential anomalies that are visually ambiguous, the module can adaptively suppress the activation of *M*(*X*), thereby assigning a larger weight (1−M(X)) to text features enriched with prior knowledge, thereby enhancing the model’s anomaly detection capability in extremely complex scenarios.

### 3.3. MIL Classification Loss

We formulate video anomaly detection as a regression problem, where the model is encouraged to assign higher anomaly scores to anomalous instances and lower scores to normal ones. The MIL-based ranking loss aims to maximize the gap between the top-scoring instances in positive and negative bags, thereby improving detection performance. However, anomalous events often consist of multiple anomalous snippets. Relying solely on the single highest-scoring instance as the optimization objective may result in missing true anomalous instances or incorrectly selecting normal ones. To address this limitation, we propose a Top-K outer bag loss, which selects the top-k highest-scoring instances from both positive and negative bags and uses their average anomaly score as the optimization signal. This strategy increases the likelihood of capturing multiple anomalous snippets within positive bags and enables the model to fully exploit the available anomaly information during training. The Top-K outer bag loss is defined as follows:

(15) $$L_{outer}=max(0,1-\frac{1}{k}\sum_{i=1}^{k}K_{max}(s_{i}^{a})+\frac{1}{k}\sum_{i=1}^{k}K_{max}(s_{i}^{n}))$$

where $K_{max}(S)$ returns the top-k elements with the highest anomaly scores $s_{i}$ from the instance-level anomaly score set *S* within a bag, while $s_{i}^{a}$ and $s_{i}^{n}$ denote the anomaly scores of segments in anomalous and normal videos, respectively.

For positive bags, the abundance of normal instances introduces significant noise, which degrades detection performance. To address this issue, we propose a k-maxmin loss within positive bags to increase the separation between instance-level anomaly scores, thereby mitigating the interference of normal instances and enhancing the model’s discriminative capability. The k-maxmin inner positive-bag loss is defined as follows:

(16) $$L_{pos}=max(0,1-\frac{1}{k}\sum_{i=1}^{k}K_{max}(s_{i}^{a})+\frac{1}{k}\sum_{i=1}^{k}K_{min}(s_{i}^{a}))$$

where $K_{min}(S)$ returns the bottom-k elements with the highest anomaly scores $s_{i}$ from the instance-level anomaly score set S within a bag.

For negative bags, hard instances with elevated anomaly scores may be present, which can hinder convergence and degrade detection performance. To address this issue, we aim to minimize the disparity of anomaly scores among instances within negative bags. Specifically, we propose a k-maxmin intra-negative-bag loss that reduces the gap between the average anomaly scores of the top-k and bottom-k instances. The k-maxmin intra-negative-bag loss is defined as follows:

(17) $$L_{neg}=L\left(B^{n}\right)=\left|\frac{1}{k}\sum_{i=1}^{k}K_{max}(s_{i}^{n})-\frac{1}{k}\sum_{i=1}^{k}K_{min}(s_{i}^{n})\right|$$

Subsequently, we integrate the k-maxmin inner positive-bag loss and inner negative-bag loss into a k-maxmin intra-bag loss, defined as follows:

(18) $$L_{inner}=L_{pos}+L_{neg}$$

We incorporate a smoothing loss [28] into the anomaly scores of instances across both positive and negative bags. This constraint is designed to enforce temporal consistency, ensuring that anomaly scores between temporally adjacent instances transition smoothly. The smoothing loss is formally defined as

(19) $$L_{smooth}=\lambda_{1}\sum_{i=1}^{T-1}\{{\left(s_{i+1}^{a}-s_{i}^{a}\right)}^{2}+{\left(s_{i+1}^{n}-s_{i}^{n}\right)}^{2}\}$$

The MIL loss function is defined as follows:

(20) $$L_{mil}=L_{outer}+\;L_{inner}+L_{smooth}$$

Finally, following the precedent set in [21], we incorporate the Binary Cross-Entropy (BCE) loss as a classification constraint to further refine the model’s discriminative ability. The total loss function is formulated as a weighted combination of the individual loss terms:

(21) $$L_{final}=\mu \cdot L_{mil}+\tau \cdot BCE(S,Y)$$

where BCE(S,Y) is the binary cross-entropy loss between the anomaly score set S and the label set Y.

## 4. Experiments

### 4.1. Dataset & Evaluation Metric

Experiments are conducted on two large-scale anomaly detection benchmarks, namely the UCF-Crime [3] and XD-Violence [6] datasets. The training and test splits of the two datasets strictly follow the official settings of UCF-Crime and XD-Violence.

UCF-Crime: This dataset comprises diverse real-world scenarios with a total duration of 128 h. It contains 13 categories of common anomalies in daily life, including 1900 untrimmed videos collected from street and indoor surveillance cameras. Both the training and test sets include normal and anomalous videos. The training set consists of 1610 videos annotated with video-level labels, while the test set contains 290 videos with frame-level annotations.

XD-Violence: This dataset contains 4754 untrimmed videos collected from multiple sources, with a total duration of 217 h. Both the training and test sets include normal and anomalous videos. The training set consists of 3954 videos annotated with video-level labels, while the test set contains 800 videos with frame-level annotations.

Evaluation Metrics: Following prior works [3,16,29], we adopt the frame-level Area Under the Receiver Operating Characteristic (ROC) Curve (AUC) as the evaluation metric on the UCF-Crime dataset. For the XD-Violence dataset, consistent with [6,20], we use Average Precision (AP) as the evaluation metric. In the context of video anomaly detection, higher AUC and AP values indicate better model performance.

### 4.2. Implementation Details

#### 4.2.1. Network Architecture

For data preprocessing, each video is uniformly divided into T = 32 temporal snippets to form the input sequence for the temporal modeling modules. Regarding the model configuration, the visual and textual backbones are initialized with the pre-trained CLIP (ViT-B/16) and remain frozen throughout the training process. To mitigate overfitting, a dropout rate of 0.6 is applied to all fully connected layers described in Section 3. Key hyperparameters are configured as follows:γ in Equation (5) is set to 1; the reduction ratio r for pointwise convolutions in Equations (11) and (12) is 32; A smaller reduction ratio r (e.g., 4 or 8) retains more channel-wise information but significantly increases the parameter count and computational cost of the pointwise convolutions, which may lead to overfitting given the limited scale of the datasets. Conversely, a larger r (e.g., 64 or 128) excessively compresses the channel dimensions, potentially causing the loss of critical semantic cues required to distinguish subtle anomalies from complex backgrounds. Therefore, we set r = 32 in this work to achieve an optimal trade-off between representational capacity and computational efficiency. The value of k in Equations (16)–(18) is set to 3; and the hyperparameter $\lambda_{1}$ in Equation (19) is 8 × 10^−5^. Furthermore, the balancing coefficients μ and τ in Equation (21) are set to 0.7 and 0.3 for the UCF-Crime dataset, and 0.01 and 1 for the XD-Violence dataset, respectively. Following VadCLIP [24], fixed textual semantic templates are adopted for text feature extraction.

#### 4.2.2. Training Procedures

The proposed TE-VTAF framework is implemented in PyTorch 1.8.1 [30] and trained on a single NVIDIA RTX 3090 GPU. The model is optimized using the Adam optimizer for 100 epochs with a batch size of 32. The initial learning rate is set to 0.001, accompanied by a weight decay of 0.005 to ensure stable convergence. To ensure reproducibility, all experiments use a fixed random seed of 42.

### 4.3. Results on Benchmark Datasets

To comprehensively evaluate the effectiveness of the proposed method, we conduct experiments on two public benchmark datasets: UCF-Crime and XD-Violence. We compare TE-VTAF with a range of representative weakly supervised approaches, including classical methods based on visual features (e.g., RTFM, MSL), approaches incorporating large language models or multimodal priors (e.g., TEVAD, VAD-CLIP, WSVAD-CLIP, RelVid), and recent architectures focusing on temporal context modeling (e.g., PECF-MIL, TCRFL). Detailed results are reported in Table 1 and Table 2.

Results on UCF-Crime: As shown in Table 1, TE-VTAF achieves the best performance on the UCF-Crime test set, reaching a frame-level AUC of 88.93%, outperforming the second-best method, VAD-CLIP [24] (88.02%), by 0.91%. Compared with early methods relying solely on visual features, TE-VTAF demonstrates substantial improvements. For instance, MIL [3] and TCN-IBL [4] achieve AUC scores of 77.90% and 78.66%, respectively, indicating that their performance is limited by single-modality representations. In contrast, our method improves performance by over 10 percentage points. Similarly, RTFM [20] and BN-SVP [31] achieve 84.30% and 83.39%, respectively, both of which are significantly outperformed by TE-VTAF. These improvements are primarily attributed to the proposed DLG-TAM module. Specifically, the LSTU effectively captures multi-scale temporal variations in abrupt anomalies, while the GTDU explicitly models long-range dependencies across snippets, enabling more expressive temporal representations than conventional static modeling or magnitude-based approaches. TE-VTAF also maintains superior performance compared with methods incorporating multi-scale or multimodal features. Sun et al. [32], Fan et al. [33], and Wang et al. [34] achieve 85.31%, 86.19%, and 84.42%, respectively, whereas our method yields improvements of 3.62%, 2.74%, and 4.51% over these approaches. This advantage is largely attributed to the VTAFM. By leveraging cross-attention, the model enables bidirectional semantic interaction between visual and textual modalities, while the Attentional Feature Fusion (AFF) mechanism dynamically adjusts their contributions. Compared with simple concatenation or attention-based fusion strategies, this design more effectively exploits complementary multimodal information and mitigates redundant noise across modalities. Furthermore, compared with other text-enhanced methods, TE-VTAF still demonstrates clear gains. For example, TEVAD [23], which fuses I3D visual features with SwinBERT textual features, achieves 84.90%, whereas our method reaches 88.93% under the same feature settings, yielding an improvement of 4.03%. This performance gap highlights the effectiveness of the AFF module, which adaptively balances visual and textual contributions based on snippet-level semantics, resulting in more discriminative cross-modal representations.

**Table 1:** Frame-level AUC (%) performance comparison on UCF-Crime.

Method	Feature	AUC (%)
MIL [3]	C3D-RGB	75.40
MIL [3]	I3D-RGB	77.90
TCN-IBL [4]	C3D-RGB	78.66
Motion-Aware [5]	PWC-Flow	79.00
GCN-AD [16]	TSN-RGB	82.12
NVAD [6]	I3D-RGB	82.44
MIST [19]	I3D-RGB	82.30
RTFM [20]	I3D-RGB	84.30
MSL [21]	VideoSwin-RGB	85.62
BN-SVP [31]	I3D	83.39
TEVAD [23]	I3D-RGB + SwinBERT	84.90
Fan et al. [33]	I3D-RGB	86.19
Sun et al. [32]	I3D	85.31
VAD-CLIP [24]	CLIP	88.02
WSVAD-CLIP [14]	CLIP	87.85
TCRFL [35]	I3D-RGB	86.76
PECF-MIL [36]	I3D	87.31
ProDisc-VAD [26]	CLIP	87.12
Wang et al. [34]	I3D-RGB + YOLOv9	84.42
RelVid [25]	CLIP	87.71
UR-DMU [37]	I3D	86.97
Motion-Prior [38]	CLIP	88.08
Ours	CLIP	88.93

Results on XD-Violence: Table 2 presents a comparison between the proposed TE-VTAF method and state-of-the-art weakly supervised anomaly detection approaches on the XD-Violence test set. The proposed method achieves an AP of 85.62%, outperforming the second-best method, PECF-MIL [35] (85.59%), by a margin of 0.03 percentage points, demonstrating its strong generalization capability in cross-domain complex scenarios. Compared with visual-feature-based methods, the conventional MIL [3] baseline achieves only 73.20%, while RTFM [20] (I3D) and MSL [21] achieve 77.81% and 78.59%, respectively. Our method substantially outperforms these approaches, further validating the effectiveness of DLG-TAM in jointly modeling local and global temporal structures via LSTU and GTDU, as well as the dynamic calibration between local and global features enabled by the BCGF mechanism. In comparison with multimodal methods, TEVAD [23] achieves 79.80%, RelVid [25] (CLIP-based) achieves 80.76%, TCRFL [36] achieves 82.63%, WSVAD-CLIP [14] achieves 83.95%, VAD-CLIP [24] achieves 84.51%, and Fan et al. [33] achieves 83.59%. The proposed method achieves the best performance at 85.62%, marginally surpassing the current state-of-the-art PECF-MIL [35] (85.59%). Notably, it outperforms VAD-CLIP and WSVAD-CLIP by 1.11% and 1.67%, respectively, further demonstrating that the VTAFM—through the joint effect of cross-attention and AFF—enables fine-grained cross-modal fusion, leading to significant improvements in feature quality under semantically complex violence detection scenarios.

**Table 2:** Frame-level AP (%) performance comparison on XD-Violence.

Method	Feature	AP (%)
MIL [3]	C3D-RGB	73.20
NVAD [6]	I3D-RGB	75.41
RTFM [20]	C3D-RGB	75.89
RTFM [20]	I3D-RGB	77.81
MSL [21]	VideoSwin-RGB	78.59
TEVAD [23]	I3D-RGB + SwinBERT	79.80
Fan et al. [33]	I3D-RGB	83.59
VAD-CLIP [24]	CLIP	84.51
WSVAD-CLIP [14]	CLIP	83.95
TCRFL [36]	I3D-RGB	82.63
PECF-MIL [35]	I3D	85.59
RelVid [25]	CLIP	80.76
UR-DMU [37]	I3D	81.66
Ours	CLIP	85.62

Overall, the proposed TE-VTAF consistently outperforms SOTA methods on two video anomaly detection benchmarks.

### 4.4. Ablation Studies

Ablation Study on Network Modules: We conduct ablation studies on multiple datasets to evaluate the effectiveness of the key components in TE-VTAF. The results are reported in Table 3 in percentage form, including AUC on UCF-Crime and AP on XD-Violence. To assess the impact of the VTAFM, we compare it with the standard concatenation (Concat) strategy. When using only raw visual features without temporal modeling (Vanilla), the model achieves 80.43% on UCF-Crime and 73.46% on XD-Violence. Introducing the Local Selective Temporal Unit (LSTU) improves the performance to 82.12% (AUC) and 75.28% (AP), indicating that multi-scale receptive fields can adaptively capture local anomalous patterns with varying temporal durations. Further incorporating the Global Temporal Distance-aware Unit (GTDU) boosts the performance to 83.26% and 78.21%, suggesting that suppressing irrelevant long-range background noise via distance-aware penalization is essential for global semantic modeling. By integrating LSTU and GTDU through the Bidirectional Cross-Gating Fusion (BCGF) branch to form the complete DLG-TAM module, the performance further increases to 84.84% on UCF-Crime and 79.07% on XD-Violence. This demonstrates that DLG-TAM effectively bridges the gap between local details and global context, leading to more precise anomaly boundary localization. Building upon the DLG-TAM-enhanced visual features, we further introduce the text modality. Using raw text features (Vanilla Text) with standard concatenation (Concat) improves performance to 86.01% (AUC) and 81.42% (AP), confirming the benefit of incorporating textual priors. Applying DLG-TAM to text features for temporal enhancement further boosts the Concat-based performance to 87.76% and 83.97%. However, simple concatenation introduces significant cross-modal noise. Replacing Concat with the Visual-Text Adaptive Fusion Module (VTAFM) leads to the best performance, achieving 88.93% AUC on UCF-Crime and 85.62% AP on XD-Violence. This result highlights the effectiveness of VTAFM: by bridging the semantic gap through mutually guided cross-attention and enabling dynamic soft selection via the AFF module, the model can adaptively balance between vision-dominant and text-dominant representations, thereby mitigating semantic inconsistency in complex surveillance scenarios.

Ablation Study on Loss Functions: We conduct ablation studies on different loss components in TE-VTAF across multiple datasets to evaluate their effectiveness. The results are reported in Table 4 in percentage form. Under the baseline setting (Ours), the model achieves 83.57% (AUC) and 79.82% (AP). Incorporating $L_{outer}$ improves the performance to 85.92% and 82.82%, respectively. This suggests that leveraging the average scores of Top-K instances enables more effective identification of multiple consecutive anomalous segments, thereby enhancing the separability between positive and negative bags. The inner bag loss consists of the inner positive loss $L_{pos}$ and the inner negative loss $L_{neg}.$ Introducing either component individually yields consistent performance gains. When combined into the full $L_{inner}$, the performance further improves to 86.12% (AUC) and 83.76% (AP). This demonstrates that the k-maxmin strategy effectively mitigates pseudo-label noise introduced by normal segments in positive bags, leading to a more compact and less noisy anomaly score distribution. Finally, jointly optimizing all proposed loss terms results in the best performance of 88.93% and 85.62%. This indicates that the inner bag and outer bag losses play complementary roles in enhancing video-level discrimination while reducing segment-level noise.

Sensitivity Analysis of Temporal Penalty Hyperparameter γ: To evaluate the robustness of TE-VTAF to the temporal penalty strength γ in the GTDU module, we conducted experiments with different values of γ ∈ {0.5, 1, 2, 5} on both UCF-Crime and XD-Violence datasets. The results are summarized in Table 5. As shown, the model performance is relatively stable across different γ values, with γ = 1 achieving the best results on both benchmarks (88.93% AUC on UCF-Crime and 85.62% AP on XD-Violence). When γ is too small (0.5), the temporal penalty is insufficient to suppress attention diffusion between temporally distant snippets, leading to slight performance degradation (−0.22% on UCF-Crime). Conversely, when γ is too large (5), the model overly suppresses long-range dependencies, reducing its ability to capture global semantic relationships critical for complex anomaly detection (−1.01% on UCF-Crime). Notably, the performance variation across γ ∈ {0.5, 2} is within ±0.3%, demonstrating that our method is robust to moderate changes in this hyperparameter. These results validate our choice of γ = 1 as an optimal balance between temporal continuity constraints and global semantic modeling.

Sensitivity Analysis of Kernel Size Selection in LSTU: To empirically validate the choice of kernel sizes in the Local Selective Temporal Unit (LSTU), we conducted an ablation study evaluating different combinations of small and large kernel branches. Specifically, we tested six configurations with kernel pairs selected from {3, 5, 7, 9}, keeping all other hyperparameters fixed. The results are summarized in Table 6. As shown, the combination (3, 7) achieves the best performance on both benchmarks (88.93% AUC on UCF-Crime and 85.62% AP on XD-Violence), while maintaining competitive computational efficiency. When the large kernel is too small (e.g., 5), the model’s capacity to capture medium-range temporal dependencies is limited, leading to slight performance degradation. Conversely, when both kernels are large (e.g., 7, 9), the increased receptive field may dilute fine-grained local anomaly signals and introduce redundant background context, resulting in marginal accuracy drops. The performance variation across configurations is within ±0.4%, demonstrating that our method is reasonably robust to kernel size selection. These results justify our choice of (3, 7) as an optimal trade-off between local detail preservation and medium-range context aggregation.

Category-Wise Cross-Modal Contribution Analysis: To further investigate the contribution of textual semantic guidance under different anomaly scenarios, we conduct a category-wise comparative analysis on the XD-Violence dataset by comparing the complete TE-VTAF framework with a visual-only variant. As shown in Table 7, the proposed visual-text adaptive fusion mechanism (VTAFM) provides varying levels of performance improvement across different anomaly categories. Specifically, the integration of textual features yields the most significant gains in Fighting (+7.59 AP) and Abuse (+7.40 AP). These categories typically involve complex human interactions, subtle behaviors, and rich contextual information, making it challenging to distinguish anomalies using visual motion patterns alone. Textual semantic priors effectively supply high-level contextual understanding, thereby enhancing the semantic discriminability of visual features. A notable improvement is also observed in Shooting (+5.32 AP). In contrast, categories such as Explosion (+1.12 AP), Riot (+1.49 AP), and Car Accident (+1.23 AP) exhibit relatively smaller improvements. This is primarily because these events contain strong, distinctive visual dynamics (e.g., intense motion, flames, or large-scale disturbances), allowing the visual branch to capture abnormal patterns effectively on its own. These observations demonstrate that the proposed VTAFM successfully exploits the complementary strengths of visual and textual modalities. Textual semantic guidance is particularly beneficial in semantically ambiguous or context-dependent anomaly scenarios, thereby improving the overall robustness and generalization capability of our framework.

## 5. Discussion

**Figure 4:**
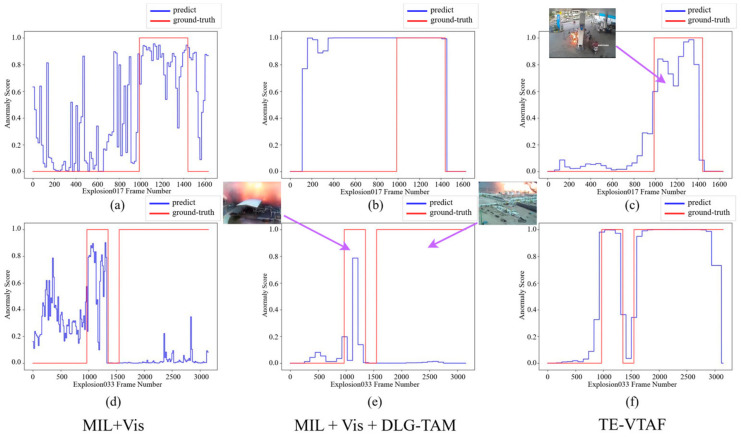
Visualization results of TE-VTAF on the UCF-Crime dataset. The blue curve denotes the predicted anomaly scores, while the red curve represents the ground-truth annotations. (**a**,**d**) Results of the baseline method using only visual features within the MIL framework. (**b**,**e**) Results after integrating the DLG-TAM module into the MIL framework, showing improved temporal alignment. (**c**,**f**) Results of the proposed full TE-VTAF model with CLIP-based visual-text fusion, demonstrating the most accurate anomaly localization.

In this section, we provide qualitative visualizations to illustrate the anomaly detection performance before and after incorporating the key components of TE-VTAF. As shown in Figure 4, the two rows present comparative results on two different anomalous videos. The first column corresponds to the baseline using visual features with the MIL framework. As shown in Figure 4a,d, relying solely on raw visual features leads to significant temporal fluctuations in the predicted anomaly scores. Notably, during event periods, a noticeable discrepancy is observed between the predicted curves and the ground-truth annotations, with frequent false positives and false negatives. The second column presents the results after integrating the DLG-TAM module into the MIL framework. As shown in Figure 4b,e, the alignment between predicted and ground-truth curves is significantly improved. The model is able to capture fine-grained anomalies and accurately localize their temporal boundaries, while temporal fluctuations are effectively suppressed. Nevertheless, false positives and false negatives still persist. The final column shows the results of the full TE-VTAF model with CLIP-based visual-text fusion. As illustrated in Figure 4c,f, the detection performance is further enhanced. This improvement can be attributed to the integration of visual and textual features, which enables a deeper understanding of high-level video semantics and leads to more accurate anomaly localization.

## 6. Conclusions

In this work, we proposed a novel Temporal-Enhanced and Visual-Text Adaptive Fusion (TE-VTAF) framework for WS-VAD in public safety scenarios. To effectively capture both short-term and long-range temporal dependencies, we first developed the DLG-TAM module to enhance local and global temporal modeling capabilities. We then introduced the VTAFM, which adaptively integrates complementary cross-modal features via a competitive activation mechanism while suppressing redundant information. Finally, we incorporated a Top-K outer bag loss and a k-maxmin inner bag loss to enhance instance-level discriminability and mitigate noise under weak supervision. Extensive experiments on two challenging WS-VAD benchmarks demonstrate the effectiveness of TE-VTAF, achieving state-of-the-art performance. In future work, we will further explore the potential of vision–language pre-trained knowledge and extend our framework to real-time video anomaly detection and open-set scenarios.

## Figures and Tables

**Table 3:** Ablation study results of network modules. DLG-TAM, LSTU, and GTDU represent using DLG-TAM, LSTU, and GTDU modules to enhance the temporal dependence between snippet features. BCGF represents the operation of using BCGF to fuse local temporal enhanced features and global temporal enhanced features. Concat and VTAFM respectively represent the fusion methods for visual and textual features.

Visual	Text	Fusion	UCF-Crime (AUC/%)	XD-Violence (AP/%)
vanilla	×	×	80.43	73.46
lstu	×	×	82.12	75.28
gtdu	×	×	83.26	78.21
dlg-tam	×	×	84.84	79.07
dlg-tam	vanilla	concat	86.01	81.42
dlg-tam	dlg-tam	concat	87.76	83.97
dlg-tam	dlg-tam	vtafm	88.93	85.62

**Table 4:** Ablation study results of the loss functions.

Method	UCF-Crime (%)	XD-Violence (%)
Ours	83.57	79.82
Ours + Louter	85.92	82.82
Ours + Lpos	84.32	81.31
Ours + Lneg	84.19	81.62
Ours + Linner	86.12	83.76
Ours + Louter + Linner	88.93	85.62

**Table 5:** Sensitivity Analysis of Temporal Penalty Hyperparameter γ.

γ Value	UCF-Crime (%)	XD-Violence (%)
0.5	88.67	85.35
1	88.93	85.62
2	88.59	85.28
5	87.92	84.71

**Table 6:** Ablation study on kernel size combinations in the Local Selective Temporal Unit (LSTU).

Kernel Pair	UCF-Crime (%)	XD-Violence (%)
(3, 5)	88.71	85.45
(3, 7)	88.93	85.62
(3, 9)	88.65	85.38
(5, 7)	88.79	85.51
(5, 9)	88.52	85.29
(7, 9)	88.34	85.12

**Table 7:** Class-wise Ablation Analysis of Cross-Modal Contributions on the XD-Violence Dataset.

Anomaly Category	Visual Only	Visual + Text	ΔAP
Abuse	56.73	64.13	+7.40
Car Accident	44.16	45.39	+1.23
Fighting	80.23	87.82	+7.59
Riot	96.12	97.61	+1.49
Explosion	73.91	75.03	+1.12
Shooting	73.05	78.37	+5.32

## Data Availability

The original contributions presented in this study are included in the article. Further inquiries can be directed to the corresponding author.
